# Supplementary material for: First record of Sigmodon minor (Rodentia) in the early Blancan of central Mexico: Asymmetrical dispersal from the Great Plains and paleoecology inferences
Source: PLoS One. 2026 Apr 9;21(4):e0346879. doi: 10.1371/journal.pone.0346879 (PMC13065024; doi:10.1371/journal.pone.0346879)
Supplement: S1 Appendix — (PDF) [file pone.0346879.s001.pdf]

Radiometric ages of the localities in the San Miguel de Allende Basin, Guanajuato, Mexico.

Radiometric dating was performed on zircon samples obtained from sediments corresponding to volcanic ash deposits interbedded with the strata containing the fossils, as previously described. These zircons were analyzed using U-Pb decay at the Laboratorio de Estudios Isotópicos of Instituto de Geociencias, Universidad Nacional Autónoma de México. The analysis was conducted through a laser ablation system coupled to an ICP-MS, focusing on the youngest and most concordant zircon samples.

The results of this analysis are presented in the concordia diagrams (S1 Fig. 1). The reported age for the GTO 6 locality is 3.68 ± 0.18 Ma, with relatively low uncertainty. This suggests an isotopic closure event in the GTO 6 samples, indicating that the dated material originated from a geologically stable environment. While the results for the GTO 12 locality, "La Pantera," report an age of 3.73 ± 0.24 Ma. Although similar to GTO 6, the higher uncertainty reflects greater data dispersion (as observed in the model, where more red ellipses indicate higher dispersion compared to the green ellipses, which represent lower dispersion). This suggests slightly discordant measurements, yet they fall within an acceptable range, validating the isotopic closure age.

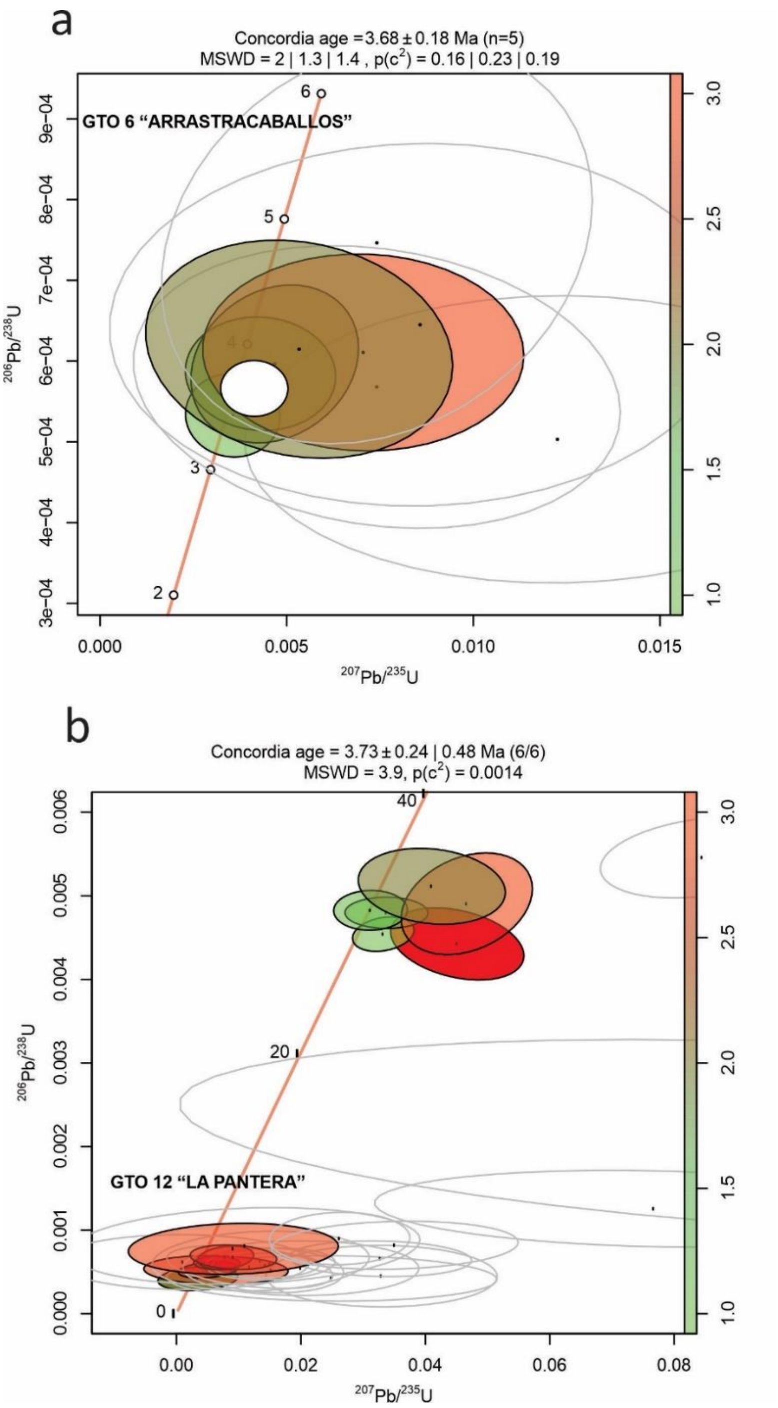

S1 Fig. 1 Radiometric ages of the localities in the San Miguel de Allende Basin, Guanajuato, Mexico: (a) locality GTO 6 “Arrastracaballos”, (b) locality GTO 12 “La Pantera”. The concordia line is represented in orange, while the ellipses correspond to the measurements for each zircon. Their size and color indicate the level of uncertainty in the data. The most concordant zircon populations, represented in green, are located closest to the concordia line. In Graph A, only one concordant population is shown for the GTO 6 locality. In contrast, Graph B reveals that for the GTO 12 locality, "La Pantera," there is evidence of an inherited zircon population. However, only the youngest population was included in determining the reported age.
