# Supplementary material for: First record of Sigmodon minor (Rodentia) in the early Blancan of central Mexico: Asymmetrical dispersal from the Great Plains and paleoecology inferences
Source: PLoS One. 2026 Apr 9;21(4):e0346879. doi: 10.1371/journal.pone.0346879 (PMC13065024; doi:10.1371/journal.pone.0346879)
Supplement: S1 Fig — (PDF) [file pone.0346879.s002.pdf]

Dental terminology

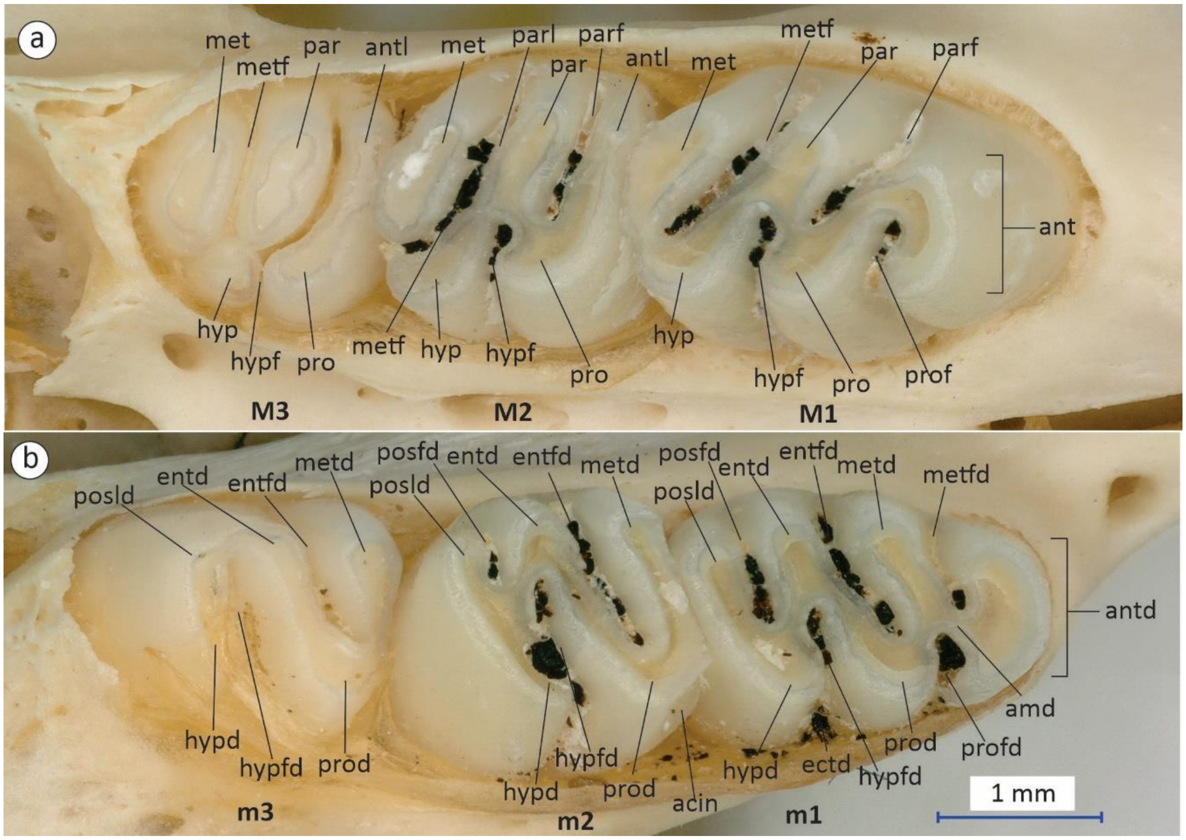

S1 Fig 1. Dental terminology used in this study illustrated with the upper and lower jaws of *Sigmodon hispidus saturatus* LACMm 18846. a) Right maxillary with the first molar (M1), second molar (M2) and third molar (M3); b) Right dentary with the first molar (m1), second molar (m2) and third molar (m3). The nterminology is based on Reig (1977) and Martin *et al* (2020). Anatomical abbreviations: **acin,** anterior cingulid; **amd,** anterior murid; **ant,** anterocone; **antd,** anteroconid; **antl,** anteroloph; **ectd,** ectostylid (structure not seen in modern *Sigmodon*); **entd,** entoconid; **entfd,** entoflexid; **hyp,** hypocone; **hypd,** hypoconid; **hypf,** hypoflexus; **hypfd,** hypoflexid; **met,** metacone; **metd,** metaconid; **metf,** metaflexus; **metfd,** metaflexid; **par,** paracone; **parf,** paraflexus; **parl,** paralophule; **posfd** posteroflexid; **posld,** posterolophid; **pro,** protocone; **prod,** protoconid; **prof,** protoflexus; **profd,** protoflexid.
