# Supplementary material for: First record of Sigmodon minor (Rodentia) in the early Blancan of central Mexico: Asymmetrical dispersal from the Great Plains and paleoecology inferences
Source: PLoS One. 2026 Apr 9;21(4):e0346879. doi: 10.1371/journal.pone.0346879 (PMC13065024; doi:10.1371/journal.pone.0346879)
Supplement: S2 Fig — (PDF) [file pone.0346879.s003.pdf]

Upper and lower molars of modern species of *Sigmodon*

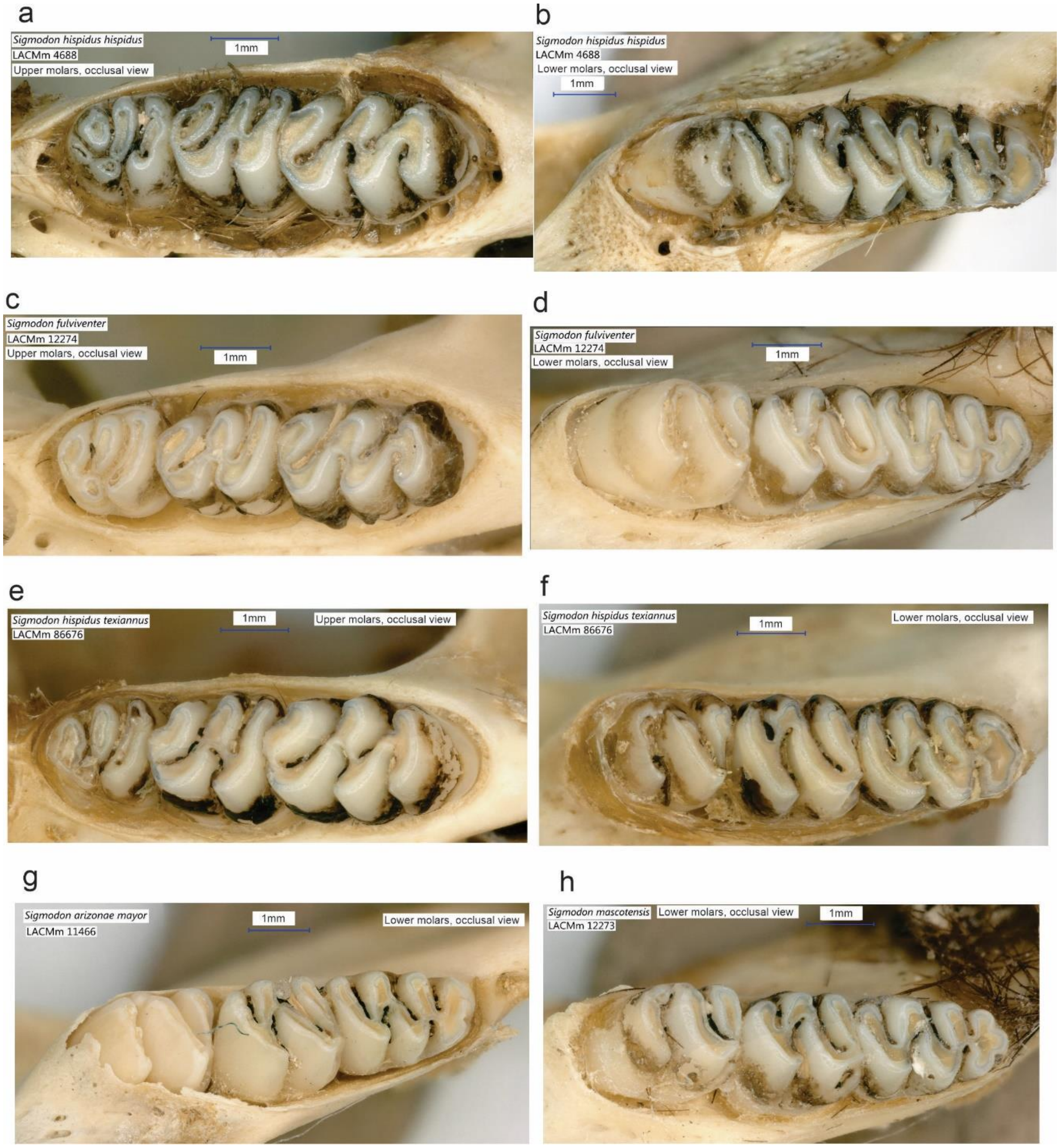

S2 Fig. Upper and lower molars of modern species of *Sigmodon*. a-b) LACMm 4688, *Sigmodon hispidus hispidus*, upper molars (a) and lower molars (b); c-d) LACMm 12274, *Sigmodon fulviventer*, upper molars (c) and lower molars (d); e-f) LACMm 86676, *Sigmodon hispidus texiannus*, upper molars (e) and lower molars (f); g) LACMm 11466, *Sigmodon arizonae*, lower molars; h) LACMm 12273, *Sigmodon mascotensis*, lower molars.
