# Supplementary material for: First record of Sigmodon minor (Rodentia) in the early Blancan of central Mexico: Asymmetrical dispersal from the Great Plains and paleoecology inferences
Source: PLoS One. 2026 Apr 9;21(4):e0346879. doi: 10.1371/journal.pone.0346879 (PMC13065024; doi:10.1371/journal.pone.0346879)
Supplement: S3 Fig — (PDF) [file pone.0346879.s004.pdf]

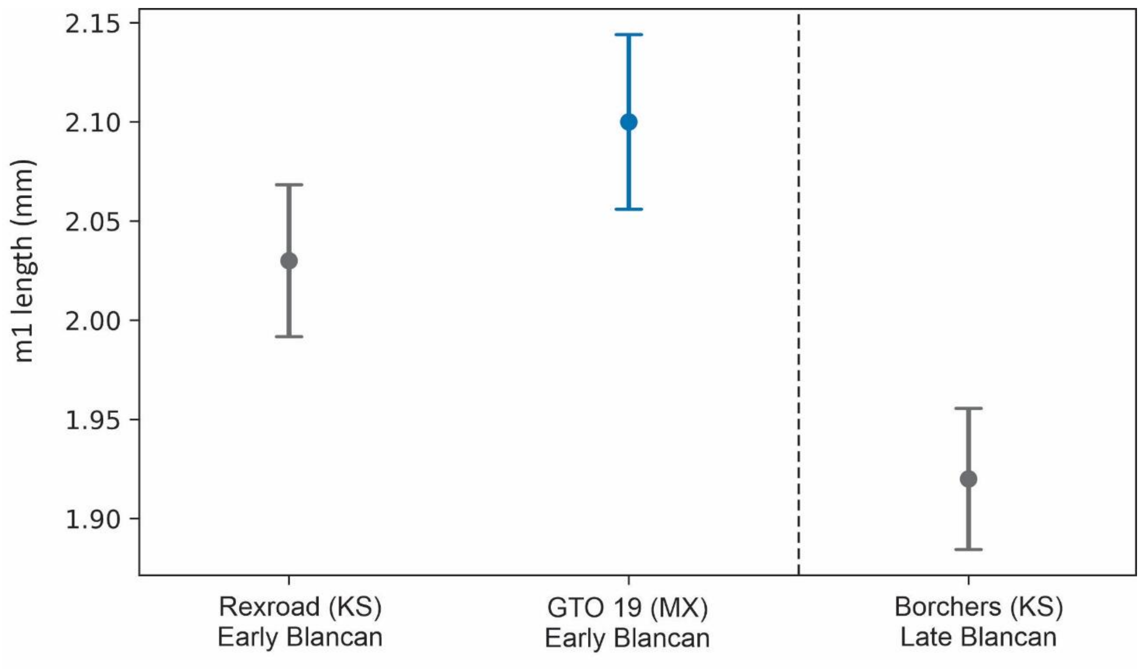

S4 Fig. Mean m1 length of *Sigmodon minor* populations from the Rexroad (Kansas), GTO 19 (central Mexico), and Borchers (Kansas) localities. Points represent group means and error bars indicate 95% confidence intervals. Rexroad (Early Blancan; N = 39) has a mean m1 length of 2.03 mm (range = 1.75–2.36 mm; SD = 0.122), GTO 19 (Early Blancan; N = 24) has a mean of 2.10 mm (range = 1.95–2.40 mm; SD = 0.11), and Borchers (Late Blancan; N = 49) has a mean of 1.92 mm (range = 1.72–2.22 mm; SD = 0.127). Localities are arranged temporally from left to right (older to younger).
