# Supplementary material for: First record of Sigmodon minor (Rodentia) in the early Blancan of central Mexico: Asymmetrical dispersal from the Great Plains and paleoecology inferences
Source: PLoS One. 2026 Apr 9;21(4):e0346879. doi: 10.1371/journal.pone.0346879 (PMC13065024; doi:10.1371/journal.pone.0346879)
Supplement: S1 Table — (PDF) [file pone.0346879.s005.pdf]

Data Provider The Paleobiology Database
Data Source The Paleobiology Database
Data License Creative Commons CC-BY
License URL http://creativecommons.org/licenses/by/4.0/
Documentation URL http://paleobiodb.org/data1.2/occs/list_doc.html
Data URL http://paleobiodb.org/data1.2/occs/list.csv?datainfo&rowcount&base_name=Sigmodon%20minor&show=genus,stratext,env,ref,refattr
Records Found 78
Records Returned 78
Records:

Occurrence_no	Collection_no	Identified_name	Accepted_name	Early_interval	Max_ma	Min_ma	Primary_reference
192941	19618	*Sigmodon medius*	*Sigmodon minor*	Blancan	4.7	1.4	N. J. Czaplewski. 1987. Middle Blancan vertebrate assemblage from the Verde Formation, Arizona. Contributions to Geology, University of Wyoming 25(2):133-155
192963	19620	*Sigmodon medius*	*Sigmodon minor*	Blancan	4.7	1.4	N. J. Czaplewski. 1987. Sigmodont rodents (Mammalia; Muroidea; Sigmodontinae) from the Pliocene (early Blancan) Verde Formation, Arizona. Journal of Vertebrate Paleontology 7(2):183-199
192969	19621	*Sigmodon medius*	*Sigmodon minor*	Blancan	4.7	1.4	N. J. Czaplewski. 1987. Sigmodont rodents (Mammalia; Muroidea; Sigmodontinae) from the Pliocene (early Blancan) Verde Formation, Arizona. Journal of Vertebrate Paleontology 7(2):183-199
192988	19624	*Sigmodon medius*	*Sigmodon minor*	Blancan	4.7	1.4	N. M. Johnson, N. D. Opdyke, and E. H. Lindsay. 1975. Magnetic Polarity Stratigraphy of Pliocene-Pleistocene Terrestrial Deposits and Vertebrate Faunas, San Pedro Valley, Arizona. Geological Society America Bulletin 86(1):5-12

193036	19628	*Sigmodon medius*	*Sigmodon minor*	Blancan	4.7	1.4	C. L. Gazin. 1942. The Late Cenozoic vertebrate faunas from the San Pedro Valley, Ariz. Proceedings of the United States National Museum 92:475-518
193110	19638	*Sigmodon medius*	*Sigmodon minor*	Blancan	4.7	1.4	N. M. Johnson, N. D. Opdyke, and E. H. Lindsay. 1975. Magnetic Polarity Stratigraphy of Pliocene-Pleistocene Terrestrial Deposits and Vertebrate Faunas, San Pedro Valley, Arizona. Geological Society America Bulletin 86(1):5-12
193124	19642	*Sigmodon medius*	*Sigmodon minor*	Blancan	4.7	1.4	N. M. Johnson, N. D. Opdyke, and E. H. Lindsay. 1975. Magnetic Polarity Stratigraphy of Pliocene-Pleistocene Terrestrial Deposits and Vertebrate Faunas, San Pedro Valley, Arizona. Geological Society America Bulletin 86(1):5-12
193144	19646	*Sigmodon minor*	*Sigmodon minor*	Blancan	4.7	1.4	N. M. Johnson, N. D. Opdyke, and E. H. Lindsay. 1975. Magnetic Polarity Stratigraphy of Pliocene-Pleistocene Terrestrial Deposits and Vertebrate Faunas, San Pedro Valley, Arizona. Geological Society America Bulletin 86(1):5-12
193182	19650	*Sigmodon minor*	*Sigmodon minor*	Blancan	4.7	1.4	J. A. Harrison. 1978. Mammals of the Wolf Ranch Local Fauna, Pliocene of the San Pedro Valley, Arizona. Occasional Papers of the Museum of Natural History, University of Kansas 73:1-18
193224	19653	*Sigmodon medius*	*Sigmodon minor*	Blancan	4.7	1.4	Y. Tomida. 1987. Small mammal fossils and correlation of continental deposits, Safford and Duncan basins, Arizona. National Science Museum
193238	19654	*Sigmodon medius*	*Sigmodon minor*	Blancan	4.7	1.4	Y. Tomida. 1987. Small mammal fossils and correlation of continental deposits, Safford and Duncan basins, Arizona. National Science Museum

193269	19656	*Sigmodon medius*	*Sigmodon minor*	Blancan	4.7	1.4	T. Galusha, N. M. Johnson, and E. H. Lindsay, N. D. Opdyke, R. H. Tedford. 1984. Biostratigraphy and magnetostratigraphy, late Pliocene rocks, 111 Ranch, Arizona. Geological Society America Bulletin 95(6):714-722
193285	19657	*Sigmodon medius*	*Sigmodon minor*	Blancan	4.7	1.4	T. Galusha, N. M. Johnson, and E. H. Lindsay, N. D. Opdyke, R. H. Tedford. 1984. Biostratigraphy and magnetostratigraphy, late Pliocene rocks, 111 Ranch, Arizona. Geological Society America Bulletin 95(6):714-722
193316	19658	*Sigmodon medius*	*Sigmodon minor*	Blancan	4.7	1.4	T. Galusha, N. M. Johnson, and E. H. Lindsay, N. D. Opdyke, R. H. Tedford. 1984. Biostratigraphy and magnetostratigraphy, late Pliocene rocks, 111 Ranch, Arizona. Geological Society America Bulletin 95(6):714-722
193343	19659	*Sigmodon medius*	*Sigmodon minor*	Blancan	4.7	1.4	Y. Tomida. 1987. Small mammal fossils and correlation of continental deposits, Safford and Duncan basins, Arizona. National Science Museum
193346	19660	*Sigmodon medius*	*Sigmodon minor*	Blancan	4.7	1.4	Y. Tomida. 1987. Small mammal fossils and correlation of continental deposits, Safford and Duncan basins, Arizona. National Science Museum
193472	19708	*Sigmodon medius*	*Sigmodon minor*	Blancan	4.7	1.4	J. Alroy. 2002. Synonymies and reidentifications of North American fossil mammals.
193520	19715	*Sigmodon medius*	*Sigmodon minor*	Blancan	4.7	1.4	J. Alroy. 2002. Synonymies and reidentifications of North American fossil mammals.
193594	19733	*Sigmodon medius*	*Sigmodon minor*	Blancan	4.7	1.4	L. B. Albright. 1999. Magnetostratigraphy and biochronology of the San Timoteo Badlands, southern California, with implications for local Plioceneâ€“Pleistocene tectonic and depositional patterns. Geological Society America Bulletin 111(9):1265-1293

193596	19734	*Sigmodon medius*	*Sigmodon minor*	Blancan	4.7	1.4	L. B. Albright. 1999. Biostratigraphy and Vertebrate Paleontology of the San Timoteo Badlands, Southern California. University of California Publications in Geological Sciences 144:1-121
193608	19736	*Sigmodon medius*	*Sigmodon minor*	Blancan	4.7	1.4	L. B. Albright. 1999. Magnetostratigraphy and biochronology of the San Timoteo Badlands, southern California, with implications for local Plioceneâ€“Pleistocene tectonic and depositional patterns. Geological Society America Bulletin 111(9):1265-1293
193623	19737	*Sigmodon medius*	*Sigmodon minor*	Blancan	4.7	1.4	L. B. Albright. 1999. Magnetostratigraphy and biochronology of the San Timoteo Badlands, southern California, with implications for local Plioceneâ€“Pleistocene tectonic and depositional patterns. Geological Society America Bulletin 111(9):1265-1293
193632	19738	*Sigmodon medius*	*Sigmodon minor*	Blancan	4.7	1.4	L. B. Albright. 1999. Magnetostratigraphy and biochronology of the San Timoteo Badlands, southern California, with implications for local Plioceneâ€“Pleistocene tectonic and depositional patterns. Geological Society America Bulletin 111(9):1265-1293
193648	19740	*Sigmodon medius*	*Sigmodon minor*	Blancan	4.7	1.4	L. B. Albright. 1999. Magnetostratigraphy and biochronology of the San Timoteo Badlands, southern California, with implications for local Plioceneâ€“Pleistocene tectonic and depositional patterns. Geological Society America Bulletin 111(9):1265-1293
193650	19741	*Sigmodon medius*	*Sigmodon minor*	Blancan	4.7	1.4	L. B. Albright. 1999. Biostratigraphy and Vertebrate Paleontology of the San Timoteo Badlands, Southern California. University of California Publications in Geological Sciences 144:1-121

193652	19742	*Sigmodon medius*	*Sigmodon minor*	Blancan	4.7	1.4	L. B. Albright. 1999. Biostratigraphy and Vertebrate Paleontology of the San Timoteo Badlands, Southern California. University of California Publications in Geological Sciences 144:1-121
193657	19744	*Sigmodon minor*	*Sigmodon minor*	Blancan	4.7	1.4	M. W. Hager. 1975. Late Pliocene and Pleistocene history of the Donnelly Ranch vertebrate fauna, southeastern Colorado. Contributions to Geology, Special Paper 2:3-62
193704	19752	*Sigmodon medius*	*Sigmodon minor*	Blancan	4.7	1.4	S. D. Webb. 1974. Chronology of Florida Pleistocene mammals. In S. D. Webb (ed.), Pleistocene Mammals of Florida
193727	19753	*Sigmodon medius*	*Sigmodon minor*	Blancan	4.7	1.4	G. S. Morgan and R. B. Ridgway. 1987. Late Pliocene (late Blancan) vertebrates from the St. Petersburg Times site, Pinellas County, Florida, with a brief review of Florida Blancan faunas. Papers in Florida Paleontology 1:1-22
194246	19925	*Sigmodon minor*	*Sigmodon minor*	Blancan	4.7	1.4	J. Alroy. 2002. Synonymies and reidentifications of North American fossil mammals.
194269	19927	*Sigmodon minor*	*Sigmodon minor*	Gelasian	2.58	1.8	J. Alroy. 2002. Synonymies and reidentifications of North American fossil mammals.
194385	19934	*Sigmodon minor*	*Sigmodon minor*	Blancan	4.7	1.4	J. Alroy. 2002. Synonymies and reidentifications of North American fossil mammals.
194401	19935	*Sigmodon minor*	*Sigmodon minor*	Blancan	4.7	1.4	J. Alroy. 2002. Synonymies and reidentifications of North American fossil mammals.
194435	19936	*Sigmodon minor*	*Sigmodon minor*	Blancan	4.7	1.4	J. Alroy. 2002. Synonymies and reidentifications of North American fossil mammals.
194460	19938	*Sigmodon medius*	*Sigmodon minor*	Blancan	4.7	1.4	M. F. Skinner and C. W. Hibbard. 1972. Early Pleistocene pre-glacial and glacial rocks and

							faunas of north-central Nebraska. Bulletin of the American Museum of Natural History 148(1):1-148
194495	19941	*Sigmodon minor*	*Sigmodon minor*	Blancan	4.7	1.4	R. E. Eshelman. 1975. Geology and Paleontology of the Early Pleistocene (Late Blancan) White Rock Fauna from North-Central Kansas. University of Michigan Papers on Paleontology 13:1-60
194520	19942	*Sigmodon minor*	*Sigmodon minor*	Blancan	4.7	1.4	R. E. Eshelman. 1975. Geology and Paleontology of the Early Pleistocene (Late Blancan) White Rock Fauna from North-Central Kansas. University of Michigan Papers on Paleontology 13:1-60
194541	19943	*Sigmodon minor*	*Sigmodon minor*	Blancan	4.7	1.4	R. E. Eshelman. 1975. Geology and Paleontology of the Early Pleistocene (Late Blancan) White Rock Fauna from North-Central Kansas. University of Michigan Papers on Paleontology 13:1-60
194571	19945	*Sigmodon minor*	*Sigmodon minor*	Blancan	4.7	1.4	R. E. Eshelman. 1975. Geology and Paleontology of the Early Pleistocene (Late Blancan) White Rock Fauna from North-Central Kansas. University of Michigan Papers on Paleontology 13:1-60
194585	19947	*Sigmodon minor*	*Sigmodon minor*	Blancan	4.7	1.4	R. E. Eshelman. 1975. Geology and Paleontology of the Early Pleistocene (Late Blancan) White Rock Fauna from North-Central Kansas. University of Michigan Papers on Paleontology 13:1-60
194706	19963	*Sigmodon medius*	*Sigmodon minor*	Blancan	4.7	1.4	P. O. McGrew. 1944. An Early Pleistocene (Blancan) fauna from Nebraska. Geological Series, Field Museum of Natural History 9(2):31-66
194981	20023	*Sigmodon medius*	*Sigmodon minor*	Blancan	4.7	1.4	W. W. Dalquest. 1975. Vertebrate Fossils From the Blanco Local Fauna of Texas.

							Occasional Papers, Museum, Texas Tech University 30:1-52
195027	20030	*Sigmodon medius*	*Sigmodon minor*	Blancan	4.7	1.4	W. W. Dalquest. 1975. Vertebrate Fossils From the Blanco Local Fauna of Texas. Occasional Papers, Museum, Texas Tech University 30:1-52
195049	20032	*Sigmodon medius*	*Sigmodon minor*	Blancan	4.7	1.4	W. W. Dalquest. 1975. Vertebrate Fossils From the Blanco Local Fauna of Texas. Occasional Papers, Museum, Texas Tech University 30:1-52
195210	20060	*Sigmodon medius*	*Sigmodon minor*	Blancan	4.7	1.4	W. W. Dalquest. 1978. Early Blancan mammals of the Beck Ranch local fauna of Texas. Journal of Mammalogy 59(2):269-298
195404	20086	*Sigmodon medius*	*Sigmodon minor*	Blancan	4.7	1.4	J. Alroy. 2002. Synonymies and reidentifications of North American fossil mammals.
195418	20093	*Sigmodon medius*	*Sigmodon minor*	Blancan	4.7	1.4	J. Alroy. 2002. Synonymies and reidentifications of North American fossil mammals.
195448	20099	*Sigmodon medius*	*Sigmodon minor*	Blancan	4.7	1.4	J. Alroy. 2002. Synonymies and reidentifications of North American fossil mammals.
195452	20101	*Sigmodon medius*	*Sigmodon minor*	Blancan	4.7	1.4	J. Alroy. 2002. Synonymies and reidentifications of North American fossil mammals.
195465	20103	*Sigmodon medius*	*Sigmodon minor*	Blancan	4.7	1.4	J. Alroy. 2002. Synonymies and reidentifications of North American fossil mammals.
195500	20106	*Sigmodon medius*	*Sigmodon minor*	Blancan	4.7	1.4	J. Alroy. 2002. Synonymies and reidentifications of North American fossil mammals.
195512	20107	*Sigmodon medius*	*Sigmodon minor*	Blancan	4.7	1.4	J. Alroy. 2002. Synonymies and reidentifications of North American fossil mammals.

195520	20108	*Sigmodon medius*	*Sigmodon minor*	Blancan	4.7	1.4	J. Alroy. 2002. Synonymies and reidentifications of North American fossil mammals.
195525	20110	*Sigmodon medius*	*Sigmodon minor*	Blancan	4.7	1.4	J. Alroy. 2002. Synonymies and reidentifications of North American fossil mammals.
195540	20112	*Sigmodon medius*	*Sigmodon minor*	Blancan	4.7	1.4	J. Alroy. 2002. Synonymies and reidentifications of North American fossil mammals.
195553	20113	*Sigmodon medius*	*Sigmodon minor*	Blancan	4.7	1.4	J. Alroy. 2002. Synonymies and reidentifications of North American fossil mammals.
195597	20116	*Sigmodon medius*	*Sigmodon minor*	Blancan	4.7	1.4	J. Alroy. 2002. Synonymies and reidentifications of North American fossil mammals.
195650	20119	*Sigmodon medius*	*Sigmodon minor*	Blancan	4.7	1.4	J. Alroy. 2002. Synonymies and reidentifications of North American fossil mammals.
195685	20120	*Sigmodon medius*	*Sigmodon minor*	Blancan	4.7	1.4	J. Alroy. 2002. Synonymies and reidentifications of North American fossil mammals.
195716	20121	*Sigmodon medius*	*Sigmodon minor*	Blancan	4.7	1.4	J. Alroy. 2002. Synonymies and reidentifications of North American fossil mammals.
195774	20123	*Sigmodon medius*	*Sigmodon minor*	Irvingtonian	1.4	0.21	J. Alroy. 2002. Synonymies and reidentifications of North American fossil mammals.
195798	20124	*Sigmodon medius*	*Sigmodon minor*	Irvingtonian	1.4	0.21	J. Alroy. 2002. Synonymies and reidentifications of North American fossil mammals.
195862	20126	*Sigmodon medius*	*Sigmodon minor*	Irvingtonian	1.4	0.21	J. Alroy. 2002. Synonymies and reidentifications of North American fossil mammals.

195907	20128	*Sigmodon medius*	*Sigmodon minor*	Irvingtonian	1.4	0.21	J. Alroy. 2002. Synonymies and reidentifications of North American fossil mammals.
196277	20176	*Sigmodon minor*	*Sigmodon minor*	Irvingtonian	1.4	0.21	R. E. Reynolds, R. L. Reynolds, and A. F. Pajak, III. 1991. Blancan, Irvingtonian, and Rancholabrean(?) land mammal age faunas from western Riverside County, California. In R.E. Reynolds, D.P. Whistler (eds.), San Bernardino County Museum Association Quarterly 38(3-4):37-40
196286	20177	*Sigmodon minor*	*Sigmodon minor*	Irvingtonian	1.4	0.21	R. E. Reynolds, R. L. Reynolds, and A. F. Pajak, III. 1991. Blancan, Irvingtonian, and Rancholabrean(?) land mammal age faunas from western Riverside County, California. In R.E. Reynolds, D.P. Whistler (eds.), San Bernardino County Museum Association Quarterly 38(3-4):37-40
196331	20186	*Sigmodon minor*	*Sigmodon minor*	Irvingtonian	1.4	0.21	A. F. Pajak, iii, E. Scott, and C. J. Bell. 1996. A review of the biostratigraphy of Pliocene and Pleistocene sediments in the Elsinore Fault Zone, Riverside County, California. PaleoBios 17(2-4):28-49
196346	20187	*Sigmodon minor*	*Sigmodon minor*	Irvingtonian	1.4	0.21	R. E. Reynolds, R. L. Reynolds, and A. F. Pajak, III. 1991. Blancan, Irvingtonian, and Rancholabrean(?) land mammal age faunas from western Riverside County, California. In R.E. Reynolds, D.P. Whistler (eds.), San Bernardino County Museum Association Quarterly 38(3-4):37-40
196377	20191	*Sigmodon minor*	*Sigmodon minor*	Irvingtonian	1.4	0.21	A. F. Pajak, iii, E. Scott, and C. J. Bell. 1996. A review of the biostratigraphy of Pliocene and Pleistocene sediments in the Elsinore Fault Zone, Riverside County, California. PaleoBios 17(2-4):28-49

196974	20298	*Sigmodon minor*	*Sigmodon minor*	Blancan	4.7	0.21	A. F. Pajak, iii, E. Scott, and C. J. Bell. 1996. A review of the biostratigraphy of Pliocene and Pleistocene sediments in the Elsinore Fault Zone, Riverside County, California. PaleoBios 17(2-4):28-49
197090	20303	*Sigmodon minor*	*Sigmodon minor*	Blancan	4.7	1.4	N. M. Johnson, N. D. Opdyke, and E. H. Lindsay. 1975. Magnetic Polarity Stratigraphy of Pliocene-Pleistocene Terrestrial Deposits and Vertebrate Faunas, San Pedro Valley, Arizona. Geological Society America Bulletin 86(1):5-12
197152	20308	*Sigmodon minor*	*Sigmodon minor*	Irvingtonian	1.4	0.21	G. S. Morgan and R. C. Hulbert, Jr. 1995. Overview of the geology and vertebrate biochronology of the Leisey Shell Pit Local Fauna, Hillsborough County, Florida. Bulletin of the Florida Museum of Natural History 37(1)
197430	20341	*Sigmodon minor*	*Sigmodon minor*	Irvingtonian	1.4	0.21	N. M. Johnson, N. D. Opdyke, and E. H. Lindsay. 1975. Magnetic Polarity Stratigraphy of Pliocene-Pleistocene Terrestrial Deposits and Vertebrate Faunas, San Pedro Valley, Arizona. Geological Society America Bulletin 86(1):5-12
197476	20344	*Sigmodon minor*	*Sigmodon minor*	Irvingtonian	1.4	0.21	N. M. Johnson, N. D. Opdyke, and E. H. Lindsay. 1975. Magnetic Polarity Stratigraphy of Pliocene-Pleistocene Terrestrial Deposits and Vertebrate Faunas, San Pedro Valley, Arizona. Geological Society America Bulletin 86(1):5-12
197737	20378	*Sigmodon cf. minor*	*Sigmodon minor*	Irvingtonian	1.4	0.21	R. E. Reynolds and R. L. Reynolds. 1994. San Bernardino County Museum Association Special Publication 94-1:31-33
295243	28221	*Sigmodon minor*	*Sigmodon minor*	Irvingtonian	1.4	0.21	H. M. Wagner and D. R. Prothero. 2001. Magnetic stratigraphy of the late Pliocene mammal-bearing deposits from Gypsum

							Ridge, San Bernardino County, California. Magnetic Stratigraphy of the Pacific Coast Cenozoic (D. R. Prothero, ed.)
295258	28223	*Sigmodon minor*	*Sigmodon minor*	Irvingtonian	1.4	0.21	H. M. Wagner and D. R. Prothero. 2001. Magnetic stratigraphy of the late Pliocene mammal-bearing deposits from Gypsum Ridge, San Bernardino County, California. Magnetic Stratigraphy of the Pacific Coast Cenozoic (D. R. Prothero, ed.)
888608	98313	*Sigmodon medius*	*Sigmodon minor*	Early Pleistocene	2.58	0.774	R. C. Hulbert. 2010. A new early Pleistocene tapir (Mammalia: Perissodactyla) from Florida, with a review of Blancan tapirs from around the state. Bulletin of the Florida Museum of Natural History 49(3):67-126
