# Supplementary material for: First record of Sigmodon minor (Rodentia) in the early Blancan of central Mexico: Asymmetrical dispersal from the Great Plains and paleoecology inferences
Source: PLoS One. 2026 Apr 9;21(4):e0346879. doi: 10.1371/journal.pone.0346879 (PMC13065024; doi:10.1371/journal.pone.0346879)
Supplement: S2 Table — (PDF) [file pone.0346879.s006.pdf]

S2 Table. Estimation of body mass (*West*) of *Sigmodon minor* from the early Blancan of San Miguel de Allende basin, Guanajuato, Central Mexico. The estimation *West* was made following Martin et al.(15), which considers the anteroposterior length in mm of the first lower molar.

#Catalog	Locality	m1 Length	*West* (gr)
MPGJ 6007	GTO 6	2.24	95.7
MPGJ1907	GTO 12	2.22	89.4
MPGJ 2650	GTO 19	2.15	75.8
MPGJ 2651	GTO 19	2.27	103.7
MPGJ 2654	GTO 19	1.95	43.0
MPGJ 2660	GTO 19	2.11	67.0
MPGJ 2669	GTO 19	2.40	142.2
MPGJ 2671	GTO 19	2.08	62.5
MPGJ 2673	GTO 19	2.06	59.0
MPGJ 2678	GTO 19	2.09	64.2
MPGJ 2681	GTO 19	2.00	49.6
MPGJ 2689	GTO 19	2.19	84.6
MPGJ 2695	GTO 19	2.15	75.6
MPGJ 2708	GTO 19	2.16	76.8
MPGJ 2709	GTO 19	2.07	59.4
MPGJ 2712	GTO 19	2.12	69.6
MPGJ 2715	GTO 19	2.40	142.2
MPGJ 2717	GTO 19	2.32	115.7
MPGJ 2725	GTO 19	2.01	50.4
MPGJ 2726	GTO 19	2.19	82.6
MPGJ 2729	GTO 19	2.22	89.7
MPGJ 2730	GTO 19	2.08	61.8
MPGJ 2731	GTO 19	2.06	58.7
MPGJ 2733	GTO 19	2.11	66.8
MPGJ 2736	GTO 19	2.22	90.9
MPGJ 2655	GTO 19	2.18	81.7
